# Aromatase excess syndromeas a model for genomic disorder: identification of molecular bases and phenotypic determinants

**DOI:** 10.1186/1687-9856-2013-S1-O20

**Published:** 2013-10-03

**Authors:** Tsutomu Ogata, Makio Shozu, Maki Fukami

**Affiliations:** 1 Department of Pediatrics, Hamamatsu University School of Medicine, Hamamatsu, Japan; 2 Department of Reproductive Medicine, Graduate School of Medicine, Chiba University, Chiba, Japan; 3 Department of Molecular Endocrinology, National Research Institute for Child Health and Development, Tokyo, Japan

## 

Aromatase excess syndrome (AEXS) is a rare autosomal dominant disorder characterized by gynecomastia. Although chromosomal inversions leading to abnormal fusion between *CYP19A1* coding exons and non-coding exons of neighboring genes have been identified in a few patients with AEXS, its molecular basis and clinical spectrum remain largely unknown. We studied 18 affected males from six unrelated families A–F, and found a heterozygous 79,156 bp tandem duplication involving seven of 11 non-coding *CYP19A1* exons 1 in families A and B, a heterozygous 211,631 bp deletion involving exons 2–43 of *DMXL2* and exons 5–10 of *GLDN* in family C, and a heterozygous 165,901 bp deletion involving exons 2–43 of *DMXL2* in families D–F. Analysis of transcripts revealed that duplicated exons 1 at the distal non-physiological position can also function as transcription start sites, and that the two deletions produced the same chimeric mRNA constituted by *DMXL2* exon 1 and *CYP19A1* exons 2–10. Clinical features such as gynecomastia and elevated estradiol/testosterone ratios were milder in patients with duplications and deletions than in those with inversions. Furthermore, genotype-phenotype correlations in patients with duplications, deletions, and inversions implies that phenotypic severity of AEXS is primarily determined by the expression pattern of *CYP19A1* and the chimeric genes and by the structural property of the fused exons with a promoter function (i.e., the presence or absence of a natural translation start codon). The present study expands the genetic mechanism and phenotypic spectrum of AEXS, and provides novel models for genomic disorder leading to gain-of-function mutations. We also discuss on the effect of estradiol on the hypothalamic-pituitary gonadotropin control.

